# Radiation therapy for oligometastatic oropharyngeal cancer

**DOI:** 10.1259/bjrcr.20190021

**Published:** 2020-02-12

**Authors:** Stefania Martini, Francesca Arcadipane, Pierfrancesco Franco, Giuseppe C. Iorio, Sara Bartoncini, Elena Gallio, Alessia S. Guarneri, Umberto Ricardi

**Affiliations:** 1 Department of Oncology, Radiation Oncology, University of Turin, Turin, Italy; 2 Department of Oncology, Radiation Oncology, A.O.U Citta’ della Salute e della Scienza, Turin, Italy; 3 Medical Physics Unit, A.O.U. Città della Salute e della Scienza, Turin, Italy

## Abstract

At presentation, isolated metastasis from oropharyngeal squamous cell carcinoma is rare. Liver is a relatively uncommon first site of failure, especially in the absence of other distant metastases, particularly without diagnosis of lung metastases. We report on a case of HPV-related oropharyngeal squamous cell carcinoma with synchronous liver metastasis treated with radiation therapy. This condition, defined as "oligometastatic state," describes a subset of patients with limited volume metastatic disease in whom favorable outcomes were reported with the use of local ablative therapies on both the primary tumor and metastatic sites. As a definitive treatment, we offered the patient, ineligible for other therapeutic approaches, exclusive radiation treatment on the head and neck region and a stereotactic ablative approach targeted to the liver metastasis.

## Case presentation

In 2016, a non-smoker 86-year-old male, affected with hypertension and Parkinson’s disease, was evaluated at the Department of Oncology of the University of Turin, due to a 6-month history of progressive odynophagia and swallowing impairment for solid food. On physical examination, a right tonsil lesion and swollen ipsilateral lymph nodes located in II level were found.

## Investigations and imaging findings

**Figure 1:**
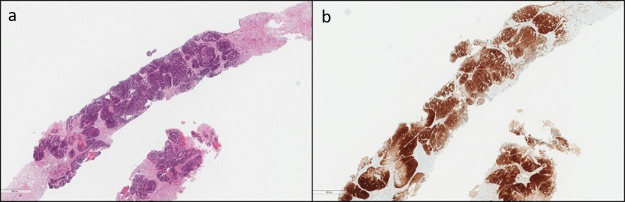
. Histological specimen OPC, hematoxylin and eosin stain. OPC, oropharyngeal carcinoma.

**Figure 2:**
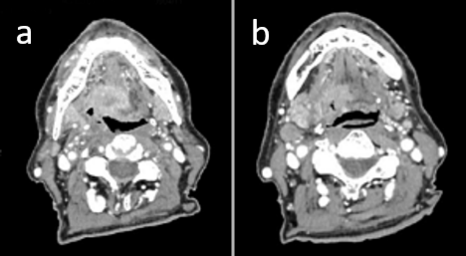
. Constrast-enhanced CT scan of primary and neck.

**Figure 3.**
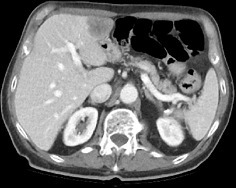
Constrast-enhanced CT scan of abdomen.

**Figure 4:**
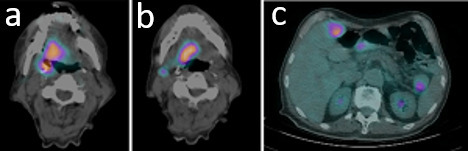
. Non-contrast positron emission tomography CT at the site of head and neck cancer and liver metastasis.

**Figure 5:**
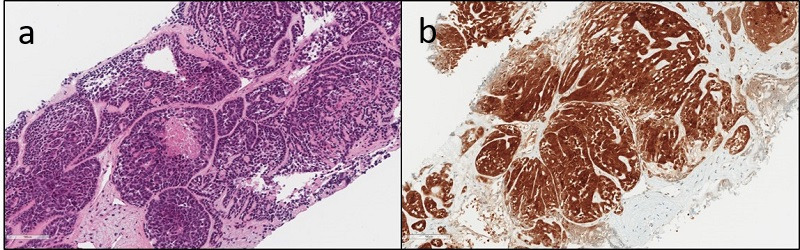
. Histological specimen of liver metastasis, hematoxylin and eosin stain.

As a part of the staging procedure a fiber-optic (FO) examination was performed with subsequent biopsies taken on the right palatine tonsil. Histological analysis revealed squamous cell carcinoma (SCC). Immunohistochemistry analysis highlighted CKAE1/AE3, EBER, p16 positivity (Figure 1). CT scan showed an infiltrative mass located to the right palatine tonsil with extension to the ipsilateral base of tongue and floor of the mouth. The overall axial plane diameter was 35 mm, 34 mm in craniocaudal axis and 23 mm in the sagittal plane (Figure 2). Cervical right lymph nodes located in II level were found (Figure 2). Furthermore, a nodular hypodense lesion (diameter 36 × 47 mm) located across the II and III hepatic segments was identified (Figure 3). Functional imaging carried out with 18-flu-2-deoxy-glucose-positron emission tomography (^18^FDG-PET) scans documented hypermetabolic uptake in left lobe of the liver with a maximum standardized uptake value (SUV max) of 12 and confirmed the locally advanced head and neck (HN) cancer (SUV max 8.8), (Figure 4). To investigate the hepatic lesion, an ultrasonography-guided Tru-Cut needle biopsy was performed with histologic diagnosis of a liver localization derived from oropharyngeal carcinoma (OPC), with a positive p16 after immunohistochemistry staining (Figure 5). In summary, the clinical tumor stage according to the eighth edition of the AJCC Cancer Staging Manual was cT2cN1cM1 (cT2cN2bcM1 according to the seventh edition of the AJCC Cancer Staging Manual) oropharyngeal cancer.

## Treatment

**Figure 6:**
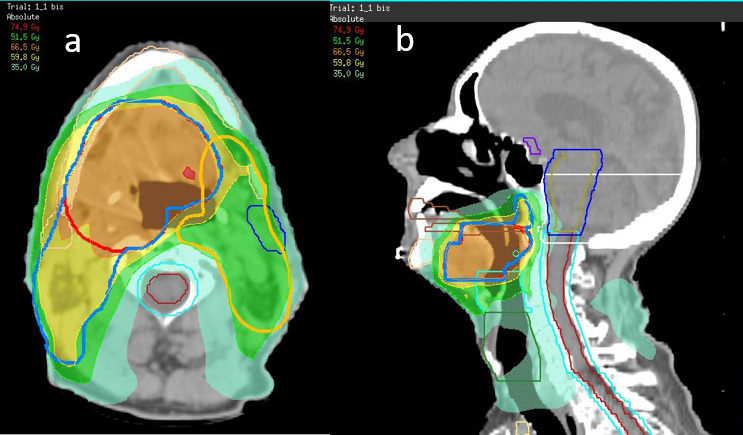
. Intensity modulated radiotherapy planning of head and neck cancer.

Taking into account the patient's age and comorbidities (ECOG PS 1), a functional assessment of swallowing (FAS) was performed and no signs of silent aspiration were observed. Moreover, a geriatric assessment was performed by Geriatric eight health status screening questionnaire. As a score of 12 was obtained (score ≤14 was defined as abnormal to the conventional classification) a comprehensive onco-geriatric evaluation was performed and an exclusive radiotherapy (RT) treatment for the HN disease was chosen.^1^ Patient had a CT simulation procedure in supine position, being immobilized with a thermoplastic mask including head and shoulders. The simulation CT scan was performed with 3 mm slice thickness axial images acquired from the vertex to the tracheal carina. An isocenter was determined within the oropharyngeal lesion and marked on the mask under laser guidance for daily setup. Gross tumor volume (GTV) comprised primary and nodal macroscopic disease. GTV was defined based on the diagnostic CT and ^18^FDG-PET information. Primary and nodal GTV was expanded isotropically by 5 mm to obtain subsequent clinical target volumes (CTV) and then optimized to avoid organs at risk (OARs). Thereafter, a 5 mm isotropic margin was added for the corresponding planning target volume (PTV) to account for setup errors and organ motion. To reduce the dose to critical structures, intensity-modulated radiotherapy (IMRT) with volumetric-modulated arc therapy (VMAT) approach was performed. Radiotherapy was administered daily, on five consecutive days per week. The prescribed dose to the primary and nodal macroscopic disease was 66 Gy/33 fractions (fr); high-risk and low-risk elective nodal PTVs received 59.4 Gy/33 fr and 51.25 Gy/33 fr, respectively. A simultaneous integrated boost (SIB) approach was employed. Monaco software version 5.11 (Elekta Oncology System^®^) was used for treatment planning and a Monte Carlo algorithm for dose calculation (Figure 6). Image-guided radiation therapy (IGRT) was performed with an automatic matching of cone-beam CT images acquired prior to each fraction delivery and reference planning CT.

Given the age and the comorbidities the patient was deemed as unfit for surgery by the liver specialists at the multidisciplinary board. Furthermore, the size of hepatic lesion did not allow for radiofrequency treatment. For the same reasons, a systemic approach was ruled out and considered as an overtreatment with more risk of toxicity enhancement than outcome benefits.

Hence, stereotactic body radiotherapy (SBRT), was chosen as a better option in order to achieve a dose escalation to the target and increase the chance of response. It was delivered concomitantly with HN IMRT during the last week of treatment. This modern RT technique enables a highly conformal dose escalation to the target improving the response to treatment, optimally sparing organs at risk.

**Figure 7.**
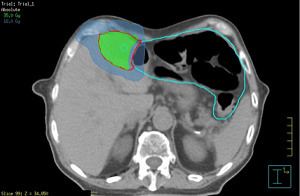
Stereotactic ablative radiotherapy planning of liver metastasis.

For the treatment of the liver metastasis, patient had a virtual simulation procedure in supine position. He was immobilized using a vacuum pillow. A four-dimensional CT was performed to define the CTV–PTV margin. The GTV included the macroscopic disease as detected on the planning CT co-registered with the diagnostic CT, employing a deformable image registration algorithm using the software Velocity (Varian Medical Systems^®^). An internal target volume (ITV) was defined to include the GTV position in all phases of the respiratory cycle. A 5 mm isotropic margin radial was added directly to ITV to create the corresponding PTV. The prescribed dose was 35 Gy in five fractions (prescription dose was set to the 80% isodose line). Treatment planning was performed using the Monaco software version 5.11 (Elekta Oncology System^®^). A Monte Carlo algorithm was employed for dose calculation (Figure 7). The treatment was delivered with an IMRT–VMAT solution (Axesse^®^ LINAC, Elekta). To verify interfraction organ motion and daily setup, IGRT was employed with a daily four-dimensional cone beam CT acquired prior to each fraction delivery, with a rigid co-registration performed and corresponding shifts acquired. All translations and rotations were corrected before treatment delivery. During the course of RT, serial clinical evaluations were performed in order to assess nutritional status and to manage adverse effects. The patient experienced acute Grade 2 oral mucositis and Grade 3 skin toxicity according the Common Terminology Criteria for Adverse Events version 4.2 classification. No hepatic toxicity was observed.

## Outcome and follow-up

**Figure 8:**
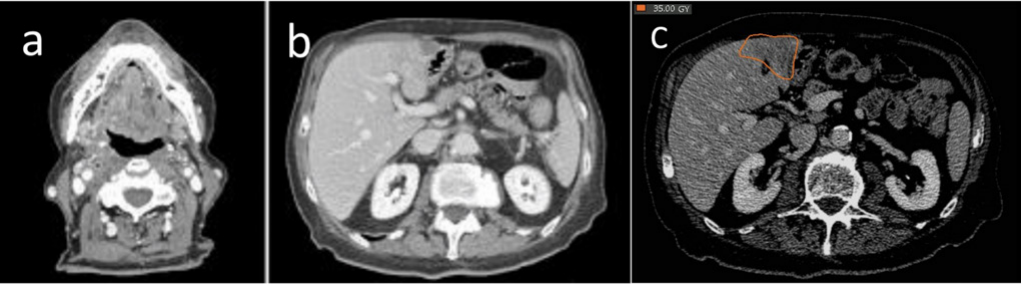
. Constrast-enhanced total body CT scan after 2 months from the end of radiotherapy. Hypodense halo, corresponding to the prescription isodose (80%) at the first CT-scan evaluation 2 months after treatment.

During the follow-up, CT-scan acquired at 2 months after the end of the treatment showed a complete response of the HN disease and a reduction in size of the hepatic metastasis (diameter 18 × 12 mm) without evidence of other tumoral sites (Figure 8). A hepatic hypodense halo, corresponding to the prescription isodose was observed at the first CT-scan evaluation 2 months after treatment, similarly as reported in a series by Howells et al (Figure 8c).^2^ No ^18^FDG-PET based restaging was performed. No visible lesions and no signs of silent aspiration were seen during FO and FAS evaluations. The next CT-scan 9 months after the end of RT, showed a stable hepatic lesion. No other metastases appeared within the liver nor elsewhere. 15 months after the end of radiotherapy the patient died for neurocognitive decline, that led to a bedridden condition and progressive elderly marasmus, without observing any frank disease progression.

## Discussion

Oropharyngeal SCC accounts for 10–12% of all HNSCC; in the western world, an increasing proportion of OPC is associated to the HPV infection, particularly in the tonsils and the base of tongue. Incidence of distant metastases in OPC is relatively low, especially in case of HPV-related (HPV+) disease. In fact, HPV+ OPC is on average less aggressive and more responsive to therapies than HPV- carcinoma.^3^ Usually OPC grows as a locally invasive disease and distant metastases occur at a later stage. At presentation, approximately 70% of patients have nodal involvement and 8% of these have distant metastases and isolated metastasis is rare.^4^ Only a limited number of studies have reported on the incidence of distant metastases from HN carcinomas at diagnosis.

Dennington et al and Black et al found distant metastases at presentation in 7 and 12% of patients with advanced-stage disease, respectively.^5,6^ About a third of patients with distant metastases presented with more than one metastatic site. The most frequent metastastic sites are lung (66%), bone (22%) and liver (10%).^4,5^ Liver is a relatively uncommon first site of failure, especially in the absence of lung metastases.^4–6^ Leemans et al reported on 26 of 281 patients with distant metastases. 21 of the 22 lung metastases were the first manifestation of distant metastases.^7^ Nishijima et al found that the lungs were not involved at autopsy in only 15% of the patients with distant metastases. In 24%, only the lungs were involved, and in 61%, the lungs were involved in combination with other sites.^8^ De Bree et al reported that 94% of the patients with systemic disease had metastases within the thorax, and all bone localizations occurred in combination with lung metastases.^9^

The oligometastatic state is conceived as an intermediate stage of disease with a prognosis comprised within those of local and widely metastatic disease, as described by Hellman and Weichselbaum in 1995.^10^ Although no definitive definition exists, the oligometastatic state is often defined as five or fewer detectable metastatic lesions in <3 organs. The implication of oligometastasis suggests that, if the primary site is resected or controlled, eradication of metastatic sites could result in better long-term disease-free interval and survival.^10^ The reported case is an unusual presentation of disease, since an isolated synchronous liver metastasis from OPC is rare. In this oncological setting, considering the single metastasis and patient’s characteristics, we administered an exclusive fractionated RT with a curative intent on the HN region and SBRT on the single liver metastasis achieving a favorable disease control with limited toxicity profile and good compliance to therapy.

The peculiarity of the radiological presentation of our case, considering the unusual site of metastatic spreading.^11^ The utilization rate of ultrasonography-guided needle biopsy has decreased in recent years. Indeed, biopsy of resectable liver metastases has been shown to be associated with a significant risk for both mechanical and oncological complications. Advances in imaging techniques allowed for a characterization of solid lesions with a diagnostic accuracy equivalent or higher than tissue biopsy and a maximum reported accuracy of 97.9%, combining contrast-enhanced ultrasound, CT and MRI, was observed.^12^ In our patient, the possibility to have a histological diagnosis on both primary tumor and metastasis, highlighted a p16 positivity on tissue specimen on both sites; the finding of this type of concordance is not frequent. However, caution has to be taken when employing the p16 protein as a sole indicator of HPV origin.^13^

Infection by high-risk genotype HPV was demonstrated to be a risk factor for the occurrence of OPC, particularly those arising from the base of tongue and tonsillar fossa in patient without significant alcohol or tobacco exposure. Prevalence of HPV+ OPC accounts for 65% of all cases in certain western countries. Patients with HPV+ OPC tend to have distinct pathological and clinical features with better overall clinical outcomes. Our case report is a perfect example of the particular HPV+ metastatic spreading pattern that, excluding the lung, is typically involving unusual sites as skin and intrabdominal lymph nodes.^11^

Moreover, HPV+ OPC are more responsive to all standard treatment approaches (surgery, RT, chemotherapy or targeted agents). Studies have demonstrated that HPV+ tumour cells are intrinsically more radiosensitive than HPV negative, due to radiation-induced sustained cell cycle arrest. New de-intensification therapeutic strategies to improve tolerability of treatment and to preserve long-term functions in OPC with favorable biology, HPV-related, have been investigated.^14–16^

In our reported case, exclusive RT was a therapeutic adaptive strategy based on the characteristics of patient and the setting of disease. A younger patient with the same disease presentation could have been approached with combining this schedule with systemic treatment. Stereotactic ablative RT allowed for an effective disease control and proved to be a safe and effective modality for the management of liver metastasis, ineligible for other local therapies. SBRT has shown in other solid tumors to fit perfectly in the oligometastatic setting management in terms of clinical outcomes and treatment-related toxicities.^17^ Trials furtherly investigating the role of SBRT as treatment of choice for metastatic sites in HN patients are warranted.

No Phase III trials present in literature compared oligometastases treated with systemic therapy alone with oligometastases treated with systemic CT and metastases directed aggressive therapy. No randomized trials compared the local therapy versus no local therapy in HN patients with lung and liver oligometastases.^18^

In the continuously changing scenario of personalized treatments tailored to the characteristics of the patient and the biological features of the disease, RT was therefore an effective treatment with a limited toxicity profile.

## Learning points

1. There exists a subset of metastatic cancer patients with limited volume metastatic disease who not only have an improved prognosis, but in whom treatment of the oligometastatic site(s) impacts on survival.
2. Isolated and synchronous liver metastasis from OPC is rare.
3. A discordance in terms of p16 positivity, at the histological work-up, between the primary and the metastatic site can be seen.
4. Stereotactic ablative radiotherapy is a potentially curative treatment.
5. Exclusive RT, as de-intensification treatment strategy, can be modulated based on the patient's characteristics, with a favorable clinical outcome, limited toxicity profile and good compliance to therapy.
6. The challenge is to identify those patients who will benefit from the treatment of their oligometastatic disease with local aggressive local therapies.
